# Analysis on the drug rules and medical thought characteristics of Six Qi prescriptions in Yuansu Zhang’s *Yixue Qiyuan*: A Review

**DOI:** 10.1097/MD.0000000000031773

**Published:** 2022-12-30

**Authors:** Yanda She, Ying Su, Yan Wang, Renhe Wang

**Affiliations:** a Changchun University of Chinese Medicine, Changchun, China; b Tonghua Normal University, Tonghua, China; c Department of Traditional Chinese Medicine, Qingdao Municipal Hospital, Qingdao University, Qingdao, China.

**Keywords:** Drug rules of Six Qi prescriptions, Medical thought, Traditional Chinese Medicine Inheritance Auxiliary Platform, *Yixue Qiyuan*

## Abstract

*Yixue Qiyuan* is a representative work of Yuansu Zhang, a famous medical scientist during the Jin Dynasty. The contents of Five Yun and Six Qi are quite rich, especially the discussion on the treatment prescription and medication of Six Qi, which has a significant impact on the development of traditional Chinese medicine. Therefore, analyzing his drug rules and summarizing his medical thoughts can provide a reference for the clinical application of the theory of Five Yun and Six Qi. Based on the TCM inheritance auxiliary platform (V2.5), the data analyzed 63 first prescriptions and 134 drugs contained in the six Qi prescription sections of *Yixue Qiyuan*. Based on the frequency analysis, correlation rule analysis, four qi and five flavors, and meridian analysis, the formula rules for wind, summer heat, wet soil, fire, dryness, and cold water were obtained, and medical thought was summarized. Drug rules of prescription and medical thought can provide a useful reference for the classical study of traditional Chinese medicine and the application of the Six Qi theory in clinical practice.

## 1. Introduction

*Yixue Qiyuan* is a representative work of Yuansu Zhang, a famous medical scientist during the Jin Dynasty. Yuansu Zhang has had a great influence on the development of Traditional Chinese Medicine (TCM). *Yixue Qiyuan* is the textbook of Yuansu Zhang’s students and the most important embodiment of his academic thoughts in his life.^[1]^

In Zhang’s medical thought, it is characteristic to attach importance to viscera syndrome differentiation and innovate the theory of drug use, and at present, the research of *Yixue Qiyuan* mostly discusses Zhang’s achievements in the theory of viscera differentiation, the theory of making prescriptions and drugs, etc.^[2–5]^ However, from a comprehensive view of the book *Yixue Qiyuan*, the contents of the Five Yun and Six Qi are quite rich, especially in the Six qi pathogenesis, Six qi treatment methods, the prescription of Six Qi treatment and medication are considered classic, and based on previous research results, this paper analyzes the drug rules of Six Qi prescriptions in *Yixue Qiyuan* based on the Traditional Chinese Medicine Inheritance Auxiliary Platform (TCMIAP) (v2.5), to more systematically analyze and summarize Zhang’s medical thoughts on Five Yun and Six Qi, and further provide valuable references for clinical practice.

## 2. Materials and methods

### 2.1. Literature source

Six types of prescriptions for wind, summer heat, wet soil, fire, dryness, and cold water were included in Yuansu Zhang’s *Yixue Qiyuan*,^[6]^ and 63 prescriptions and 134 medicines were selected.

### 2.2. Prescription selection and standardization

A prescription with a clear name, indication, and drug composition shall be taken as the inclusion criteria.

The names of drugs were standardized according to *Pharmacopoeia of the People’s Republic of China*^[7]^ and *Traditional Chinese Pharmacology*.^[8]^ For example, Chijian is standardized as Tianma, Maren as Huomaren, Liangjiang as Gaoliangjiang, Gange as Gegen, etc.

### 2.3. Prescription input and analysis method

#### 2.3.1. Input method.

Apply the “Prescription management” module in the “Platform management” of the TCMIAP (v2.5) jointly developed by the Institute of TCM of the Chinese Academy of Sciences and the Institute of Automation of the Chinese Academy of Sciences to input prescriptions that met the inclusion criteria. To ensure the accuracy of the input prescriptions, a specially assigned person proofreads them again after the input.

#### 2.3.2. Analysis method.

The statistical report system and data analysis system of the TCMIAP (v2.5) were used to analyze the frequency, association rules, four Qi and five flavors, meridian tropism, and other aspects of the six Qi prescriptions for wind, summer heat, wet soil, fire, dryness, and cold water in *Yixue Qiyuan*.

## 3. Results

### 3.1. Frequency analysis

Of the six Qi prescriptions, 12 prescriptions of wind were included and 88 drugs were used; there were 10 prescriptions for summer heat and 28 drugs in total, there were 9 prescriptions in the category of wet soil, 28 drugs were used; fire category included 11 prescriptions, 32 drugs were used; the dryness category included 10 prescriptions, 32 drugs were used; there were 11 cold water prescriptions and 21 drugs. Table 1 shows the top 10 results of the frequency of administration for the six types of prescriptions.

**Table 1:** Frequency of six qi prescription drugs.

Wind		Summer heat		Wet soil		Fire		Dryness		Cold water	
Drugs	Frequency	Drugs	Frequency	Drugs	Frequency	Drugs	Frequency	Drugs	Frequency	Drugs	Frequency
Gancao	10	Gancao	9	Baizhu	5	Dahuang	9	Dahuang	8	Ganjiang	10
Chuanxiong	8	Renshen	5	Huashi	5	Gancao	8	Huomaren	5	Gancao	7
Fangfeng	7	Baizhu	4	Rougui	4	Huangqin	4	Taoren	3	Fuzi	6
Renshen	6	Fuling	4	Zhuling	4	Mangxiao	4	Qianghuo	3	Baizhu	5
Danggui	5	Banxia	3	Muxiang	4	Zhizi	3	Muxiang	3	Gaoliangjiang	3
Dahuang	4	Huashi	3	Zexie	4	Houpo	2	Renshen	2	Renshen	3
Zhusha	4	Shigao	3	Chenpi	3	Huanglian	2	Gancao	2	Rougui	3
Tianma	4	Zexie	2	Chifuling	3	Zhiqiao	2	Houpo	2	Banxia	2
Qianghuo	4	Huoxiang	2	Fuling	3	Baishao	2	Danggui	2	Caodoukou	2
Bohe	4	Gegen	2	Tinglizi	3	Huashi	2	Xingren	2	Biba	2

### 3.2. Association rule analysis

The function of “formula rule” was used to conduct the correlation rule analysis of Six qi prescriptions. The drug patterns are shown in Tables 2 and 3.

**Table 2:** Drugs use mode of wind, summer heat and wet soil.

Wind		Summer heat		Wet soil	
Drug mode	Frequentness	Drug mode	Frequentness	Drug mode	Frequentness
Chuanxiong, Gancao	7	Renshen, Gancao	5	Baizhu, Zhuling	4
Chuanxiong, Fangfeng	6	Baizhu, Gancao	4	Baizhu, Zexie	4
Gancao, Danggui	5	Gancao, Fuling	4	Baizhu, Rougui	4
Chuanxiong, Gancao, fangfeng	5	Baizhu, Fuling	4	Zexie, Rougui	4
Chuanxiong, Renshen	4	Baizhu, Gancao, Fuling	4	Baizhu, Zexie, Rougui	4
Chuanxiong, Bohe	4	Renshen, Baizhu	3	Huashi, Baizhu	3
Renshen, Gancao	4	Renshen, Fuling	3	Huashi, Zhuling	3
Renshen, Fangfeng	4	Renshen, Baizhu, Fuling	3	Huashi, Muxiang	3
Gancao, Bohe	4	Renshen, Baizhu, Gancao	3	Chenpi, Huashi	3
Gancao, Dahuang	4	Renshen, Gancao, Fuling	3	Baizhu, Fuling	3

**Table 3:** Drugs use mode of fire, dryness and cold water.

Fire		Dryness		Cold water	
Drug mode	Frequentness	Drug mode	Frequentness	Drug mode	Frequentness
Gancao, Dahuang	7	Dahuang, Huomaren	5	Gancao, Ganjiang	6
Mangxiao, Dahuang	4	Taoren, Dahuang	3	Fuzi, Ganjiang	5
Huangqin, Dahuang	3	Qianghuo, Dahuang	3	Gancao, Fuzi	5
Mangxiao, Gancao	3	Dahuang, Muxiang	3	Baizhu, Gancao	5
Mangxiao, Gancao, Dahuang	3	Danggui, Dahuang	2	Baizhu, Ganjiang	4
Huangqin, Zhizi	2	Gancao, Dahuang	2	Gancao, Fuzi, Ganjiang	4
Huangqin, Gancao	2	Dahuang, Zhishi	2	Baizhu, Gancao, Ganjiang	4
Huangqin, Bohe	2	Dahuang, Kezi	2	Ganjiang, Rougui	3
Huangqin, Huanglian	2	Dahuang, Yuliren	2	Ganjiang, Gaoliangjiang	3
Zhizi, Gancao	2	Dahuang, Rougui	2	Renshen, Ganjiang	3

### 3.3. Analysis of four qi and five flavors and their meridian tropism

The Four Qi and Five flavor statistics and meridian statistics functions were used to analyze the six qi prescriptions. The results are presented in Tables 4 and 5, respectively.

**Table 4:** Analysis of Four Qi and Five flavors table of Six Qi prescriptions.

Prescription type	Four Qi (Including mild)					Five flavors				
	Cold	Heat	Warm	Cool	Mild	Sour	Bitter	Sweet	Pungent	Salty
Wind	36	3	68	5	30	4	52	64	84	9
Summer heat	15	0	20	3	15	1	16	40	19	2
Wet soil	18	5	22	0	9	0	24	31	23	1
Fire	33	0	9	2	9	2	35	19	11	4
Dryness	16	2	25	0	15	6	40	24	26	0
Cold water	0	25	19	0	8	1	11	25	35	0

**Table 5:** Drug meridian analysis of six *qi* prescriptions.

Prescription type	Meridian tropism											
	Liver	Heart	Spleen	Lung	Kidney	Stomach	Gallbladder	Large intestine	Small intestine	Bladder	Trijoule	Pericardium
Wind	59	51	65	62	21	53	13	18	6	29	0	0
Summer heat	3	24	37	30	10	36	4	4	0	7	2	0
Wet soil	7	12	30	22	20	26	5	10	0	18	4	0
Fire	19	19	31	26	0	34	8	24	8	7	0	9
Dryness	24	16	38	16	11	28	6	34	0	3	0	8
Cold water	3	30	45	28	22	36	0	5	0	0	0	0

## 4. Discussion

The results were obtained by data mining 63 prescriptions and 134 drugs contained in the Six *Qi* Prescriptions of *Yixue Qiyuan* through the TCMIAP (v2.5). This paper discusses the rules of drugs and the medical thought embodied in them.

### 4.1. The drug rules of wind prescriptions

In this kind of high-frequency drugs, Chuanxiong, Fangfeng, Tianma, Qianghuo and Bohe belong to the category of wind growth and rise in *Yixue Qiyuan*, These drugs are thin in taste, belong to the yang within yin, have the property of pungent, dispersing heat and promoting, can dispel wind, disperse cold, clear away heat and remove dampness, and are the main drugs for Zhang’s treatment of wind. Zhang’s experience in treating rheumatism was inherited and developed by his disciple Dongyuan Li, who called it the medicine of wind in his works. It is often used to treat internal and miscellaneous diseases.^[9]^ Gancao known as the “national elder,” has the function of harmonizing various drugs and alleviating drug properties in prescriptions, which is one of the reasons for its high frequency of use and high correlation with other drugs in the statistics of various prescriptions.^[10–22]^ Gancao also has the functions of invigorating the spleen and Qi, clearing away heat and toxin, eliminating phlegm, relieving cough and relieving pain,^[8]^ and its role in wind prescriptions is slow down with sweetness. Renshen is a tonic for Qi, which can greatly replenish vital energy, restore pulse and solidify detachment, replenish spleen and lungs, generate fluid and nourish blood, calm nerves and benefit intelligence; Danggui is a blood tonic. Chuanxiong promotes qi and blood circulation. Renshen, Danggui, and Chuanxiong are used in wind power prescriptions to strengthen health. When combined with other drugs to treat wind, it means “treating blood before treating wind, self-eliminating of wind after blood circulation “. Dahuang can attack accumulation and clear away heat and fire, Zhusha is a kind of calming drug, and *Yixue Qiyuan* says that “the heart heat cannot be eliminated unless it is used.”^[23–28]^ Wind evils are often associated with fire and heat. Dahuang, Zhusha and other high-frequency drugs are used as exorcism drugs in wind prescriptions, and their work is to expel the evil of fire and heat.

According to the four qi and five flavors of wind prescriptions and their meridian tropism, the five flavors are mainly pungent, sweet, and bitter; the four qi are mainly warm, cold, flat, and cool; and the meridian tropism is mainly the spleen, lung, liver, stomach, and heart meridian. *Zhizhenyaodalun* says: “the wind evil in the interior, treat with pungent and cool, supplement with bitter, ease with sweet, and dispel with pungent.” The four qi and five flavors of wind prescriptions embody the six-qi therapy. Most wind-induced dizziness belongs to the liver, the lung dominates the skin, and the heart is on the surface. External winds attack the human body from its surface. Therefore, wind prescriptions are mostly used for the liver, lungs, and heart meridians. Most drugs for returning the spleen and stomach embody the idea of preventing diseases by seeing the liver, knowing that the liver transmits the spleen and strengthens the spleen first.

### 4.2. The drug rules of summer heat prescriptions

Among these high-frequency drugs, Gancao, Renshen, Baizhu, Banxia, and Huoxiang belong to the category of “moistening into the center” in *Yixue Qiyuan*, which mainly refers to the spleen and stomach of the human body. Fuling, Huashi, and Zexie belong to the categories of dryness, descent, and convergence, which can facilitate urination. Shigao is cold, with a cold qi, pungent, and sweet taste. It is an important drug for the treatment of fever, manic fever, diurnal tidal fever, spontaneous sweating, cloudy and red urine, thirst, and body muscle fever. Gegen is a wind-rising type, which can eliminate the deficiency heat of the spleen, stomach, and thirst. According to the drug combination obtained from the association rules, it is the drug composition of the Sijunzi decoction. The Sijunzi decoction comes from *Taipinghuiminhejijufang*, which is a classic recipe for invigorating the middle qi and strengthening the spleen and stomach. The evil qi of summer heat injures the middle energizer, causing abnormal transmission, vomiting, diarrhea, and thirst. Renshen, Baizhu, and Gancao tonified the middle Jiao and Qi, strengthened the spleen and stomach, and generated body liquids. Fuling is used to dehumidify and diuresis, providing a way to evil the Qi of summer heat. The summer heat prescriptions based on Sijunzi decoction or its drug combination, such as Guiling Ganlu drink, Guiling Baizhu powder, Baizhu powder, Xiaochaihu Decoction, etc., all mean this.

According to the Four Qi and Five flavors of summer heat prescriptions and their meridian tropism, the four qi are mainly warm, cold, and flat; the five flavors are mainly sweet, pungent, and bitter, supplemented by salty and sour, which reflects the Six Qi therapy of *Zhizhenyaodalun*. Heat and lust are in the interior, cured by salty and cold, supplemented by sweet and bitter, astringent with sour, and diffuse with bitter. The meridians were mainly the spleen, stomach, lung, and heart meridians. *Yixue Qiyuan* summarizes the pathogenesis of summer heat is “such as asthma, vomiting, acid, diarrhea, cramping, muddy urine, abdominal distension and swelling, carbuncle, ulcer, rash, tumor qi tuberculosis, vomiting cholera, depression and swelling, nasal strangulation, blood overflow and bleeding, body heat, chills, sadness and laughter, delirium, all belong to heat.” The spleen, stomach, lung, and heart are the main sites of injuries caused by summer heat evil Qi, so the drugs prescribed mostly belong to the above meridians.

### 4.3. The drug rules of wet soil prescriptions

In this kind of high-frequency drugs, Baizhu and Chenpi belong to the category of dampness in *Yixue Qiyuan*, which can strengthen the spleen and stomach, promote qi stagnation and eliminate dampness. Huashi, Zhuling, Zexie, and Fuling belong to the category of dryness; they are those with thin Qi. They are the yin within yang, can facilitate urination, and give outlets to dampness evil. Rougui and Muxiang belonged to the heat category. They are those with thick Qi. They are Yang within the Yang. Dampness is the evil of Yin, and it is easy to suppress the Qi mechanism and damage the Yang Qi. Warm drugs, such as Rougui and Muxiang, are used to invigorate Yang Qi and promote stagnation to dispel evil. Tinglizi can relieve lung and asthma and swelling.^[8]^ It is used to treat edema and asthma caused by dampness, abdominal distension, adverse urination, and other syndromes. According to the drug combination obtained from the association rules, Baizhu with Zhuling, Zexie, Rougui, and Fuling constitute the Wuling powder and Guilingbaizhu pill. Wuling powder is the classic prescription for the treatment of water dampness in *Shanghanlun*, and the Guilingbaizhu pill is *Xuanminglunfang*, which has the advantages of eliminating phlegm, stopping cough, and dispersing fullness and congestion. Huashi, Baizhu, Zhuling, Muxiang, and Chenpi are important components of the Tinglimuxiang powder, Baizhumuxiang powder, and Dajupi decoction. These three prescriptions were all derived from *Xuanminglunfang* to treat swelling, asthma, and cough caused by damp heat.

According to the four Qi, five flavors, and meridian tropism of the wet soil prescriptions, the four Qi are mainly warm, cold, and flat, and the five flavors are mainly sweet, bitter, and pungent. It should be noted that there is no “light” flavor in the five flavor statistics of the auxiliary platform for the inheritance of TCM. However, according to the *Pharmacopoeia of the people’s Republic of China*^[7]^ and the textbook of *TCM*,^[8]^ Huashi, Zhuling, Zexie, and Fuling have sweet and light flavors. Therefore, wet soil prescriptions reflect the Six Qi therapy of *Zhizhenyaodalun* “wet is in the interior, and it is treated with bitter heat, supplemented with salty and light, with bitter dryness, and with light discharge.” The main meridians are the spleen, stomach, lungs, kidneys, and bladder. *Yixue Qiyuan* summarizes that the main treatment preparation belongs to the pathogenesis of rheumatism as “most kinds of spasms and rigidity, accumulated fluid and fullness in the septum, cholera vomiting, swollen, meat like mud, can not afford to press them, most belong to dampness.” The spleen, stomach, lung, kidney, and bladder are mainly injured by evil dampness, so the drugs are mostly based on the above meridians.

### 4.4. The drug rules of fire prescriptions

In this kind of high-frequency drug, Dahuang, Huangqin, Mangxiao, Zhizi, and Huanglian belong to the cold category in *Yixue Qiyuan*; they are thick in flavor and Yin within Yin. These drugs have cold *Qi* and a bitter flavor, and can eliminate the evil of fire and heat. Gancao is used to clear away heat and poison and to reconcile various drugs to ease the drug potential and prevent the bitter cold from purging the medicine too much and damaging the healthy qi.^[29–38]^ Houpo and Zhiqiao Wide middle focus, moving qi and eliminate swelling in Sanyichengqi Decoction and Dachengqi decoction. Baishao replenishes the middle energizer and blood vessels. Huashi has cold qi and sweet and light flavor, it can cure the red and astringent urination caused by fire evil. According to the drug combination obtained from the association rules, Dahuang, Gancao and Mangxiao constitute Tiaoweichengqi Decoction, which is an important component of Liangge powder, SanyiChengqi Decoction and Taoren Chengqi Decoction, and the combination of Huangqin, Dahuang, Huanglian and Zhizi is an important component of HuanglianJiedu decoction.

According to the four Qi and five flavors of fire prescriptions and their meridian tropism, the four Qi are mainly cold, warm, and flat, and the five flavors are mainly bitter, sweet, and pungent, which reflect the Six Qi therapy of *Zhizhenyaodalun*: “the fire is in the interior, the treatment is salty and cold, accompanied by bitter and pungent, astringent with sour and diffuse with bitter.” The main meridians are the stomach, spleen, lungs, large intestine, heart, and liver. *Yixue Qiyuan* concludes the pathogenesis of fire, which is “most kinds of heat, sudden ignorance, rashness, anger, swearing, horror, swelling, pain and sour, qi rushing upward, shudder-like loss of consciousness, sneezing, vomiting, sore throat, tinnitus or deafness, vomiting overflowing, unable to eat, unclear vision, sudden diarrhea, sudden death from violent illness, all belonging to fire.” The stomach, spleen, lung, large intestine, heart, and liver meridians are the main sites of injury caused by fire evil, so the drugs are mostly based on these meridians.

### 4.5. The drug rules of dryness prescriptions

In this kind of high-frequency drug, Dahuang is bitter and cold, it can clean the intestines and stomach, eliminate dryness of the yangming, and promote the old to the new. Huomaren, Taoren, and Xingren have moistened dryness. Huomaren can moisten the intestines, relieve constipation, and tonify deficiencies. For example, the PIyue pill takes it as a king medicine to moisten the dryness of the intestines. Taoren is moist and is used to treat blood knots. Xingren moistened lung qi and removed lung dryness. Muxiang can cause Qi flow and dispel stagnation, while Houpo can remove abdominal distension. Qianghuo is pungent, bitter, and mild, and is used in combination with Dahuang and other bitter cold drugs to treat wind heat and dryness, moistening the dryness with pungency, and reducing it with bitterness, such as Runchang pill, Maren pill, and Qisheng pill. Renshen, Gancao, Danggui, and other drugs are used to replenish qi and blood and are designed to strengthen the body. High-frequency drug combinations are a combination of Dahuang and moistening dryness, promoting Qi, expelling wind, and other drugs to moisten dryness and save dryness.

According to the four Qi and five flavors of dryness prescriptions and their meridian tropism, the four Qi are mainly warm, cold, and flat, and the five flavors are bitter, pungent, and sweet, which reflects the Six Qi therapy of *Zhizhenyaodalun*: “dryness and adulteration are treated with bitter and warm, supplemented with sweet and pungent, moistened with bitter, and reduced with bitter.” The main meridians are the spleen, large intestine, and stomach. “Medical Qiyuan” concludes that the main treatment preparation of the Internal Classic belongs to dryness, and the pathogenesis is “all astringent dryness and vigorous chapping belong to dryness.” The spleen, stomach, and large intestine meridians are mainly injured by dryness evil; therefore, the prescriptions are mostly based on the above meridians.

### 4.6. The drug rules of cold water prescriptions

In this type of high-frequency drug, Ganjiang, Fuzi, Gaoliangjiang, Rougui, and Caodoukou are classified as heat and growth in *Yixue Qiyuan*; they are thick in Qi and Yang within Yang. Biba was pungent and hot. These hot drugs are usually King drugs in cold prescriptions. Gancao, Baizhu, Renshen, and Banxia are examples of dampness transformations. These drugs can replenish the qi of the spleen and stomach in the middle energizer and remove dampness and phlegm. These are usually official or assistant drugs. The high-frequency drug combination of Gancao-Ganjiang, Fuzi-Ganjiang, Gancao-Fuzi, and Gancao-Fuzi-Ganjiang is composed of the Sini decoction, Jiangfu decoction, and Jiajian Baitong decoction. Fuzi and Ganjiang were hot and pungent, respectively. Fuzi walks but does not stop, penetrating up, down, inside, and outside, and Ganjiang keeps but does not go; it can stick to the nature of Fuzi and enhance its ability to warm the middle and return to the Yang.^[39–48]^ Gancao is beneficial to Qi and concordant middle-jiao, and can slow down the nature of dryness and fierceness of Ganjiang and Fuzi. Baizhu-Gancao, Baizhu-Ganjiang, Baizhu-Gancao-Ganjiang, and Renshen-Ganjiang are composed of Lizhong and Fuzi Lizhong pills. Baizhu is sweet and warm, can strengthen the spleen and stomach, remove dampness, and remove heat in the stomach. Renshen is warm and beneficial to Qi, and plays the role of removing cold dampness and regulating middle-jiao with Gancao and Ganjiang. The combination of Ganjiang, Rougui, and Gaoliangjiang forms the composition of the Dajihan pill. Rougui is used to supplement the lack of heat in down-jiao, and Gaoliangjiang is used to remove the cold in the stomach. The combination of Ganjiang and Rougui was used to remove the accumulated cold.

According to the four Qi and five flavors of cold prescriptions and their meridian tropism, the four Qi are mainly heat and warm, and the five flavors are mainly pungent, sweet, and bitter, which reflects the Six Qi therapy of *Zhizhenyaodalun*: “the cold is in the interior, the treatment is sweet and hot, accompanied by bitter and pungent, spread it with pungent and firm it with bitter.” The meridians are mainly spleen, stomach, heart and lung meridians. *Yixue Qiyuan* concludes the pathogenesis of cold, which is “the clear and cold water from the upper and lower parts of various diseases, the tumor, hernia, blocked and hard, the full and acute pain of abdomen, diarrhea, no hungry, vomiting, inconvenient flexion and extension, and the reverse cooling, forbidden and solid, all belong to cold.” The spleen, stomach, heart, and lung meridians are mainly injured by cold evil, so prescription drugs are mostly based on the above meridians.

### 4.7. Characteristics of medical thought

#### 4.7.1. Observe the pathogenesis of six qi and differentiate flexibly.

Yuansu Zhang’s *Yixue Qiyuan* elucidated the pathogenesis of six qi through a special article on six qi for disease and six qi for disease interpretation.^[10]^ Six Qi prescriptions were used to treat the six Qi pathogeneses. For example, the wind prescriptions, such as Shenxianhuangu pill, Huashexuming decoction, Fangfeng Tianma powder, Qufeng pill, Datongshengbaihuashe powder, are aimed at the violent rigidity, pain, softness and contraction of tendons in the wind pathogenesis; Baihu Decoction, a prescription for summer heat, aims at the body heat that belongs to the pathogenesis of fever, and Guiling Ganlu drink and Guiling Baizhu powder aim at vomiting cholera; The Tingli Muxiang powder, Baizhumuxiang powder and Dajupi decoction of wet soil prescriptions are aimed at the accumulated fluid, fullness in the septum, heavy body and swelling.

The use of the six Qi prescriptions reflects the characteristics of Yuansu Zhang’s prescription with flexible syndrome differentiation. For example, the treatment methods for stroke are different: those with visceral diseases should sweat first when the disease is on the surface; those with visceral diseases should relieve their bowels first and then pass through the meridians. There are two ways to treat wind: to regulate meridians and blood, and diaphoresisl. “Fangfeng Tongsheng powder, Lingsha pill, Shenxianhuangu pill, Fangfeng Tianma powder We considered the external and internal wind heat. Jiajianchonghe decoction and Datongshenghuashe powder were used to treat wind in hollow organs. Huomingjindan treats wind in the organs. Medication with syndrome is common in prescriptions, such as the Jiajianchonghe decoction, for those who sweat a lot, add Huangqi, for those who cough, and add Wuweizi. Zhang believes that ancient and modern Qi, geographical environment, and climate have different effects on human life activities and diseases. Therefore, when ancient prescriptions are used to treat diseases, they should not remain the same. The prescription should be used flexibly according to time and local conditions.

#### 4.7.2. Observe the drug rules and distinguish the generation and restraint among five elements.

Yuansu Zhang divided drugs into five categories according to the thickness of the odor and the ups and downs in *Yixue Qiyuan*. In addition, according to these five elements, a method of making prescriptions, such as the wind method, was established. Liver, wood, and acid are also the means of spring life. If they are abnormal, they become ill. Wind internally, governance with pungent and cool, bitter and pungent, sweet and slow, and pungent. Based on the theory of Qi in the Internal Classic of Huang Di, Yuansu Zhang absorbed and brought into play Wansu Liu ‘s theory of six evils and diseases,^[11]^ emphasizing that the key to the formulation of drugs based on four Qi and five flavors is to follow the change law of Yin and Yang in nature and the nature of the rise and fall of the visceral Qi mechanism. At the same time, he should also follow the principle of “the higher suppresses it and the lower raises it in the Internal Classic of Huang Di, His formulation and medication should study the effects of drugs on exogenous wind from the perspective of four Qi and five flavors of drugs and the overall rise and fall of Yin and Yang, and it is believed that the direction of drug action is multidimensional.

#### 4.7.3. Embody the theory of syndrome differentiation of viscera and meridians.

The theory of differentiation of viscera and meridians is an important part of Zhang Yuanyuan’s medical thought, The six Qi prescriptions of *Yixue Qiyuan* embody the above two aspects.^[49–51]^ For example, Fangfengtongsheng powder, a wind-type prescription, can cure stagnation of heat accumulation in the intestines and stomach, deficiency of kidney water and Yin, heat storms of heart fire and yang, and even stroke. Lingshadan can treat gastrointestinal dryness and astringency. Jiajianchonghe decoction and Datongshenghuashe powder were used to treat wind in hollow organs. Huomingjindan cures the viscera in the wind. Zhibaodan can cure heat accumulation in the heart and lungs, and constipation in the large intestine. The chifuling pill, a wet soil prescription, can cure excessive dampness in the spleen and stomach. It can be seen from the statistical results of the six Qi prescriptions that they embody a clear theory of drug channel tropism.

## 5. Conclusion

Based on the TCMIAP (v2.5), this study analyzed the data of the prescriptions contained in the six Qi prescriptions in *Yixue Qiyuan*. According to the frequency analysis, association rule analysis, four Qi and five flavors, meridian analysis, the drug rules of various prescriptions, and the medical thought embodied in them are summarized to provide a useful reference for the classical research of TCM and better use Yuanwu Zhang ‘s theory of five movements and six Qi to guide clinical practice.

## Author contributions

**Data curation:** Yan Wang.

**Project administration:** Yanda She, Ying Su, Renhe Wang.

**Writing – original draft:** Yanda She.

**Writing – review & editing:** Ying Su.
